# Variations among *Streptococcus gallolyticus* subsp. *gallolyticus* strains in connection with colorectal cancer

**DOI:** 10.1038/s41598-018-19941-7

**Published:** 2018-01-24

**Authors:** Ritesh Kumar, Jennifer L. Herold, John Taylor, Juan Xu, Yi Xu

**Affiliations:** Center for Infectious and Inflammatory Diseases, Institute of Biosciences and Technology, Texas A&M Health Science Center, Houston, TX USA

## Abstract

*Streptococcus gallolyticus* subsp. *gallolyticus* (*Sg*) has long been reported to display a strong association with colorectal cancer (CRC). It was recently demonstrated to actively promote the development of CRC, underscoring the importance of *Sg* in both clinical correlation and functional relevance in CRC. Here we investigated several clinical isolates of *Sg* in their interactions with human colon cancer cells and in mouse models. Some *Sg* strains were able to stimulate host cell proliferation (proliferation-promoting *Sg*, PP-*Sg*) whereas others were not (non-proliferation-promoting *Sg*, NP-*Sg*). PP-*Sg* strains adhered to colon cancer cells much better than NP-*Sg* strains, suggesting that close contact between *Sg* and host cells is important. In mice, PP-*Sg* is significantly better at colonizing the colon tissues of A/J mice compared to NP-*Sg*, however this difference was not observed in C57BL/6 mice, suggesting that *Sg* colonization of mouse colon tissues involves specific interactions between bacterial and host factors on the colonic epithelium. Finally, in an azoxymethane-induced mouse model of CRC, PP-*Sg* promoted tumor development whereas NP-*Sg* did not. These findings provide clues to the mechanism underlying the *Sg*-CRC association and have important implications to clinical studies that aim to correlate *Sg* with clinical and pathological features of CRC.

## Introduction

Intestinal microbes have a profound influence on the health and disease status of the human body. Colorectal cancer (CRC) is one of the diseases that have been shown to be modulated by microbes^1–10^. This modulation raises the possibility that by incorporating the contributions of microbes, more optimized strategies for cancer prevention and treatment may be developed. To realize this potential, a better understanding of the specific details of the microbe-CRC connection is important.

*Streptococcus gallolyticus* subsps. *gallolyticus* (*Sg*), previously known as *Streptococcus bovis* biotype I, is an opportunistic human pathogen that causes bacteremia and endocarditis. Numerous studies over the last several decades have described elevated risks for CRC in patients infected with *S. bovis* or *Sg*^1,4,5,11–25^. For example, a study by Boleij *et al*. reviewed 52 case reports and 31 case series published in PubMed up to 2011 and found that among *S. bovis*-infected patients who underwent colonic evaluation, ~60% had concomitant colorectal adenoma/adenocarcinoma^13^, much higher than that in the general population. The *S. bovis* group includes several bacterial species. Among the different species, patients with bacteremia/endocarditis due to *Sg* have the strongest association with CRC (~7 fold higher risk compared to those with infections caused by other species in the *S. bovis* group^13^, suggesting *Sg*-specific mechanisms are involved in the *Sg*-CRC connection.

Recent studies found that CRC patients with no symptoms of active infections can be “silently” infected with *Sg*^10,26^. Although the specific frequencies vary among the studies possibility due to the different detection methods used and different geographical locations where the samples were collected, common themes emerge - *Sg* is found in substantial percentages of CRC patients (up to 74%), much higher than in healthy individuals^22,26^, and preferentially associates with tumor tissues compared to adjacent normal tissues. Taken together, clinical and epidemiological studies clearly support a strong correlation between *Sg* and CRC.

In addition to the strong clinical correlation, recent results showed that *Sg* also actively promotes the development of CRC^10^. *Sg* stimulates colon cancer cell proliferation via the β-catenin signaling pathway, enhances tumor growth in a xenograft model and promotes colon tumor development in an azoxymethane (AOM)-induced mouse model of CRC. The increased cell proliferation and elevated β-catenin signaling was observed in *in vitro* cultured cells, in xenografts, and in colonic crypt cells in the mouse model. Moreover, two recent studies followed patients with endocarditis due to *Sg* for a number of years and found that ~23–45.2% of patients with *Sg* endocarditis developed new colonic neoplasm during the follow-up period^27,28^. On the other hand, patients with endocarditis due to other pathogens showed a much lower rate of having new colonic neoplasm^27,28^. Taken together, these results support the notion that *Sg* may play a causal role in CRC. Thus, it seems that *Sg* not only has a strong correlation with CRC but also is functionally involved in the development of CRC. Further investigations are clearly needed in order to understand the effects of *Sg* in more specific and detailed manner.

In this study, we examined a number of *Sg* clinical isolates for their effect on cell proliferation and tumor development. The results divide the isolates into two subgroups - one able to stimulate host cell proliferation and promote colon tumor development, and the other not. We also investigated the ability of *Sg* strains to adhere to colon cancer cells and to colonize the mouse colon. The results suggest that there are strain-specific mechanisms involved in the interaction between *Sg* and the colonic epithelium, and in its ability to promote colon tumor development. This is the first report on the variations among *Sg* isolates in connection with CRC. The results suggest that future studies to investigate the relationship between *Sg* and CRC not only need to distinguish *Sg* at the species level but also at the strain level. The correlation between the ability to adhere and colonize colon tissues and the ability to promote host cell proliferation and tumor growth suggests close interactions between *Sg* and the colon epithelium is important for *Sg* to exert its influence on tumor development.

## Results

### Promotion of human colon cancer cell proliferation by *Sg* is strain specific

**Figure 1:**
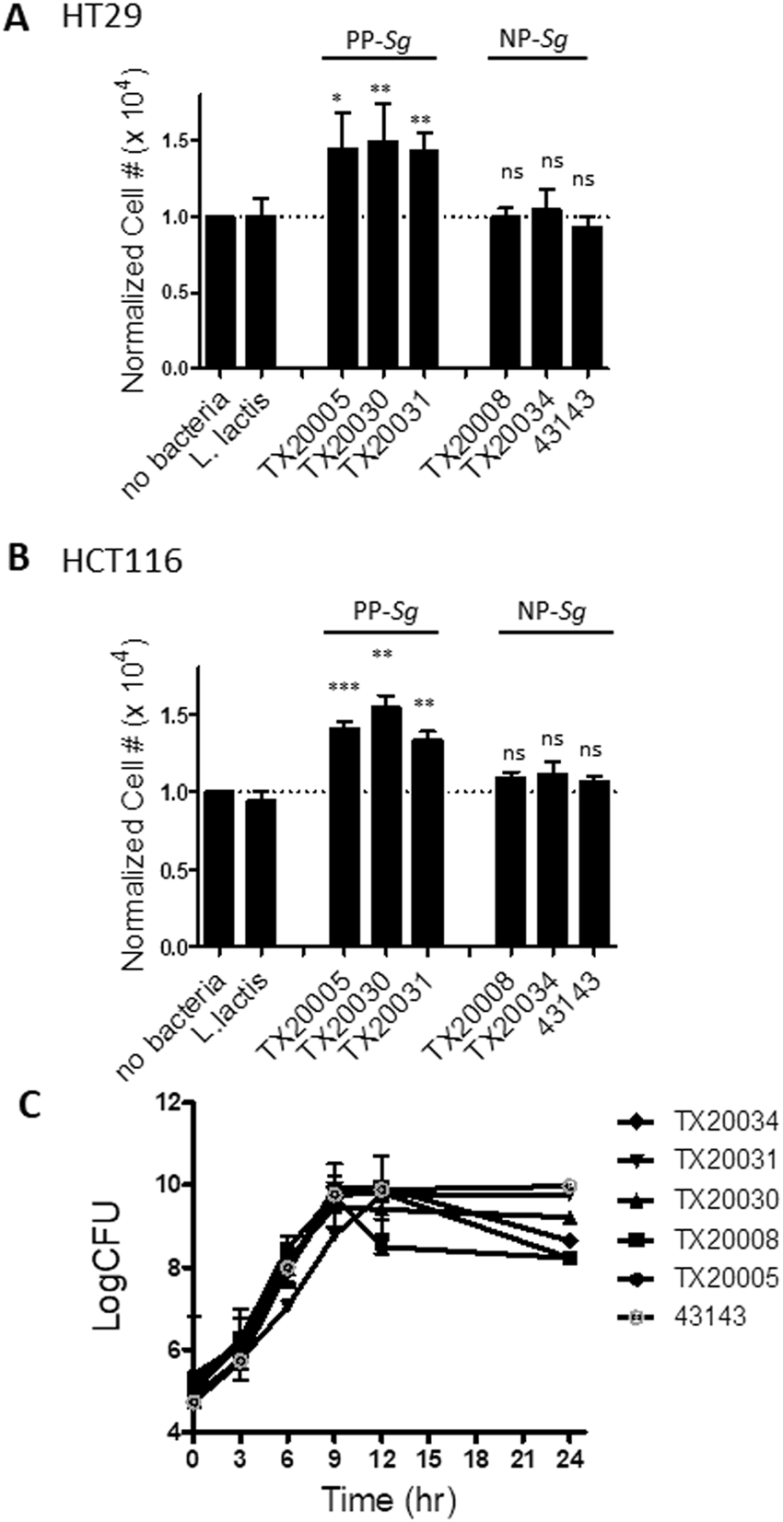
Variations among *Sg* clinical isolates in their ability to stimulate host cell proliferation. (**A** and **B**) Cell proliferation assays. HT29 (**A**) and HCT116 (**B**) cells were co-cultured with *Sg* strains TX20005, TX20030, TX20031, TX20008, TX20034 or ATCC 43143, *L. lactis*, or media only for 24 hours and viable cell numbers enumerated. The results shown were combined from at least three independent experiments, each done with two technical replicates. *p < 0.05; **p < 0.01; ***p < 0.001, unpaired two-tailed *t* test, vs. no bacterial control. (**C**) The growth curves of the *Sg* strains. *Sg* strains were cultured in BHI broth at 37 °C with shaking. Samples were taken from the cultures at indicated time points and dilution plated onto agar plates to determine the bacterial counts. The results shown were combined from three independent experiments.

We examined a number of *Sg* strains isolated from the blood of endocarditis patients^29^ for their effect on host cell proliferation. HT29 and HCT116 cells were co-cultured with *Sg* strains TX20005, TX20030, TX20031, TX20008, TX20034, and ATCC 43143 in tissue culture plates for 24 hours. Viable cells were enumerated in an automated cell counter. *Lactococcus lactis* was used as a negative bacterial control. The results showed that TX20005, TX20030 and TX20031 were able to promote cell proliferation, consistent with previous results^10^. Surprisingly, co-culture with TX20008,TX20034 and ATCC 43143 had no effect on cell numbers in either HT29 (Fig. 1A) or HCT116 cells (Fig. 1B). The *Sg* strains showed similar growth curves (Fig. 1C), indicating that the inability of TX20008, TX20034 and ATCC 43143 to stimulate host cell proliferation was not due to any defect in bacterial growth. Thus, the former group of *Sg* strains (TX20005, TX20030 and TX20031) is referred to as “proliferation-promoting *Sg*” (PP-*Sg*), and the latter as “non-proliferation-promoting *Sg*” (NP-*Sg*) for the rest of the study.

**Figure 2:**
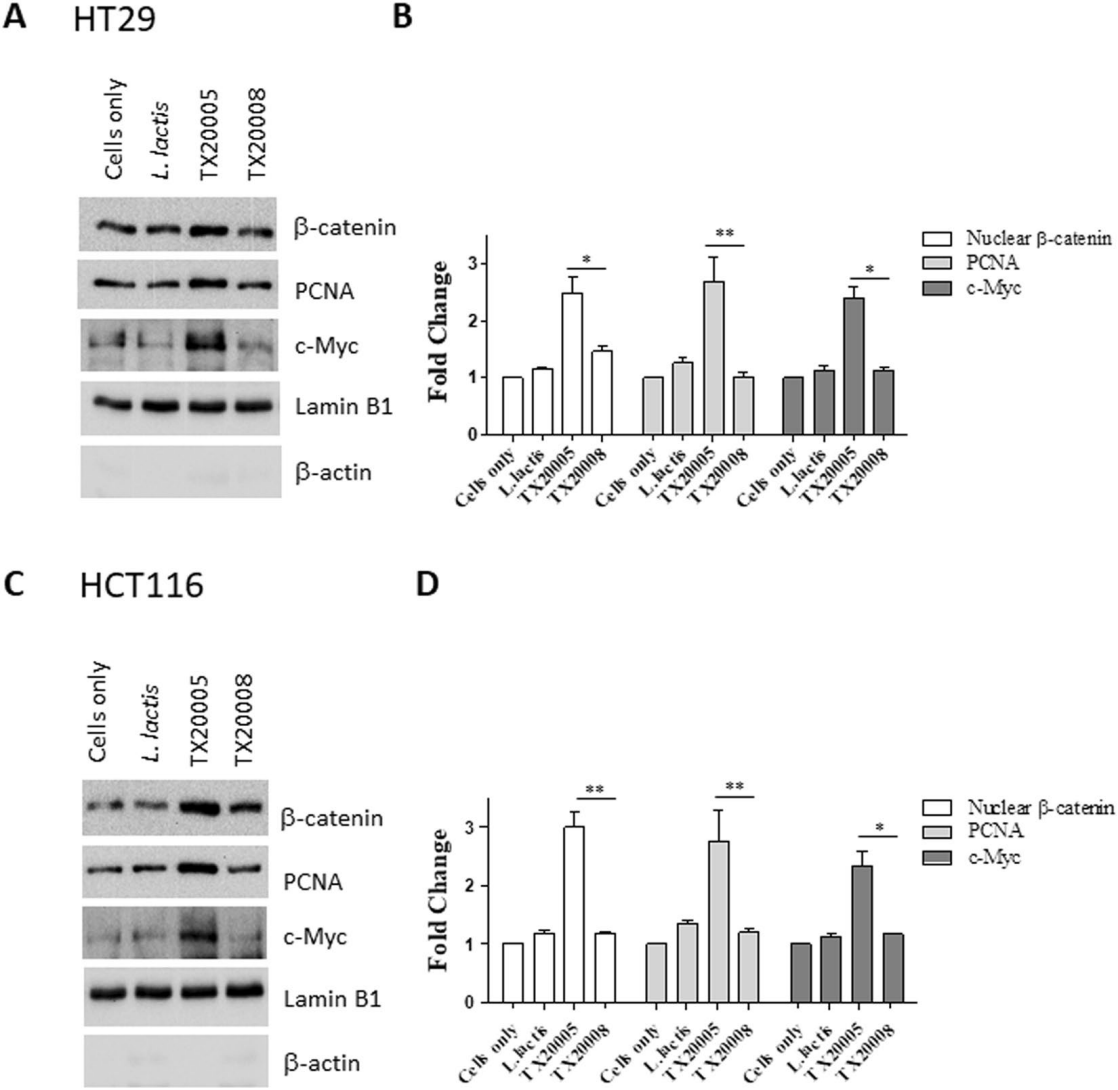
PP-*Sg* and NP-*Sg* differ in their ability to up-regulate cell proliferation markers. HT29 (**A** and **B**) and HCT116 (**C** and **D**) cells were co-cultured with PP-*Sg* strain TX20005, NP-*Sg* strain TX20008, *L. lactis* or media only as described in the Methods section. Nuclear extracts were subjected to SDS-PAGE and western blot. Representative images are shown (**A** and **C**). Band intensities were measured using Image J and normalized to that of lamin B1 first, and then to cells only control. Data shown were combined from at least three independent experiments (**B** and **D**). *p < 0.05; **p < 0.01; unpaired two-tailed *t* test, TX20005 vs. TX20008 treatment.

Previously it was reported that TX20005 promoted colon cancer cell proliferation via the β-catenin signaling pathway. Co-culture with TX20005 led to increased expression of β-catenin, c-Myc, and proliferating cell nuclear antigen (PCNA) in HT29 and HCT116 cells^10^. Therefore, we examined the effect of PP-*Sg* strains TX20030 and TX20031,- and NP-*Sg* strains TX20008, TX20034 and ATCC 43143 on the expression of these molecules. Similar to TX20005, TX20030 and TX20031 increased the expression of β-catenin, c-Myc and PCNA. On the other hand, TX20008, TX20034 and ATCC 43143 had no effect on the protein level of β-catenin, c-Myc or PCNA (Fig. 2A–D, Supplemental Fig. 1), further confirming the inability of the NP-Sg strains to stimulate host cell proliferation.

We previously showed that the ability to promote host cell proliferation is dependent on bacterial growth-phase. Stationary phase TX20005 and TX20030 were able to stimulate cell proliferation, whereas exponential phase bacteria were not^10^. We performed experiments to test if exponential phase NP-*Sg* strains TX20008, TX20034 and ATCC 43143 were able to stimulate cell proliferation. The results showed that they were not (Supplemental Fig. 2), suggesting that the inability of the NP-*Sg* strains to stimulate host cell proliferation was not due to differential regulation of relevant *Sg* factors by growth phase, but rather an absence or inactivity of the *Sg* factors in this group of strains.

### NP-*Sg* strain TX20008 does not promote tumor growth in a xenograft model

**Figure 3:**
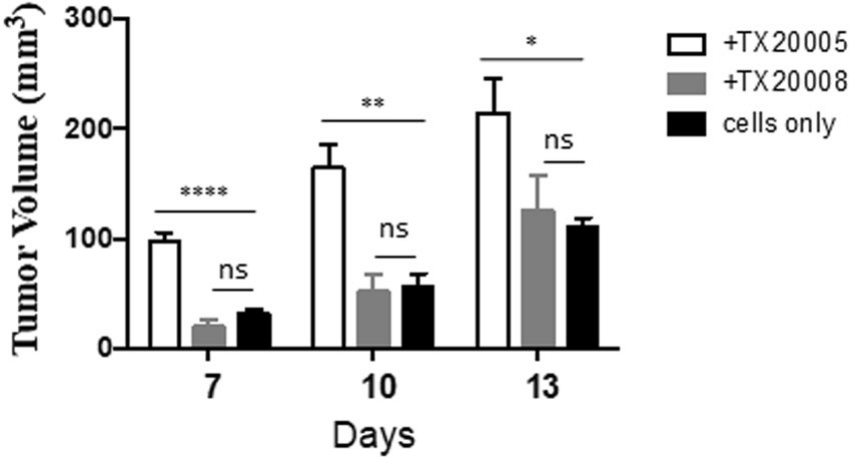
NP-*Sg* strain TX20008 does not promote tumor growth in a xenograft model. HCT116 cells were co-cultured with TX20005, TX20008, or media only as described in the Methods section. The cells were mixed with Matrigel and injected into nude mice (5 mice/group). Tumor size was monitored at indicated time points. *p < 0.05; **p < 0.01; ****p < 0.0001; unpaired two-tailed *t* test.

To further examine the ability of PP-*Sg* and NP-*Sg* to stimulate cell proliferation *in vivo*, we co-cultured HCT116 cells with TX20005, TX20008 or media only. The cells were then mixed with Matrigel and injected into nude mice. Tumor formation and growth was monitored. The results showed that cells pre-exposed to TX20005 formed significantly larger tumors compared to untreated cells at day 7, 10, 13 post injection. In contrast, tumors formed by cells pre-exposed to TX20008 showed no difference compared to those from untreated cells (Fig. 3), suggesting that TX20008 is also unable to stimulate cell proliferation *in vivo*.

### Adherence of *Sg* strains to human colon cancer cells

**Figure 4:**
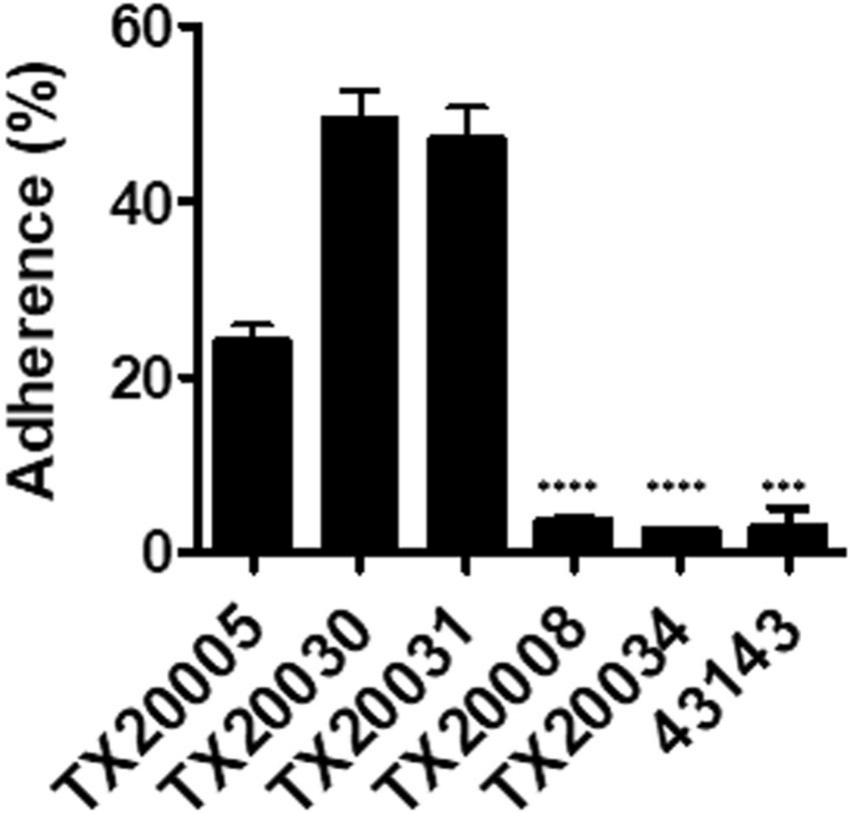
NP-*Sg* strains adhere to human colon cancer cells less effectively than PP-*Sg* strains. HT29 cells were incubated with overnight cultures of the various *Sg* strains (MOI = 10) for 1 hour, washed, lysed and diluted plated. Adherence was calculated as the percentage of recovered bacteria over total bacteria added to each well. The results shown were combined from three independent experiments. ***p < 0.001; ****p < 0.0001; unpaired two-tailed *t* test, vs. TX20005.

We compared the ability of stationary PP-*Sg* and NP-*Sg* strains to adhere to HT29 cells. All three PP-*Sg* strains adhered to the cells significantly better than the NP-*Sg* strains (Fig. 4). The number of adhered bacteria as a percentage of total bacteria added is 24.1 ± 1.8% for TX20005, 49.4 ± 3.4% for TX20030, 47.0 ± 3.8% for TX20031, 3.6 ± 1.3% for TX20008, 2.5 ± 0.3% for TX20034 and 2.8 + 1.3% for ATCC 43143. These results suggest that there are variations among *Sg* strains with respect to their ability to adhere to human colon cancer cells and the adherence capability appears to correlate with their capacity to stimulate cell proliferation.

### PP-*Sg* specific factors contribute to gut colonization in A/J mice

Next, we examined the ability of PP-*Sg* strain TX20005 and NP-*Sg* strain TX20008 to colonize mouse colon. A/J mice were orally gavaged with TX20005 and TX20008, respectively. Colon tissues and fecal materials were collected at day 3 and 7 post gavage to determine the *Sg* concentration using a qPCR protocol that specifically detects *Sg*^10^.

**Figure 5:**
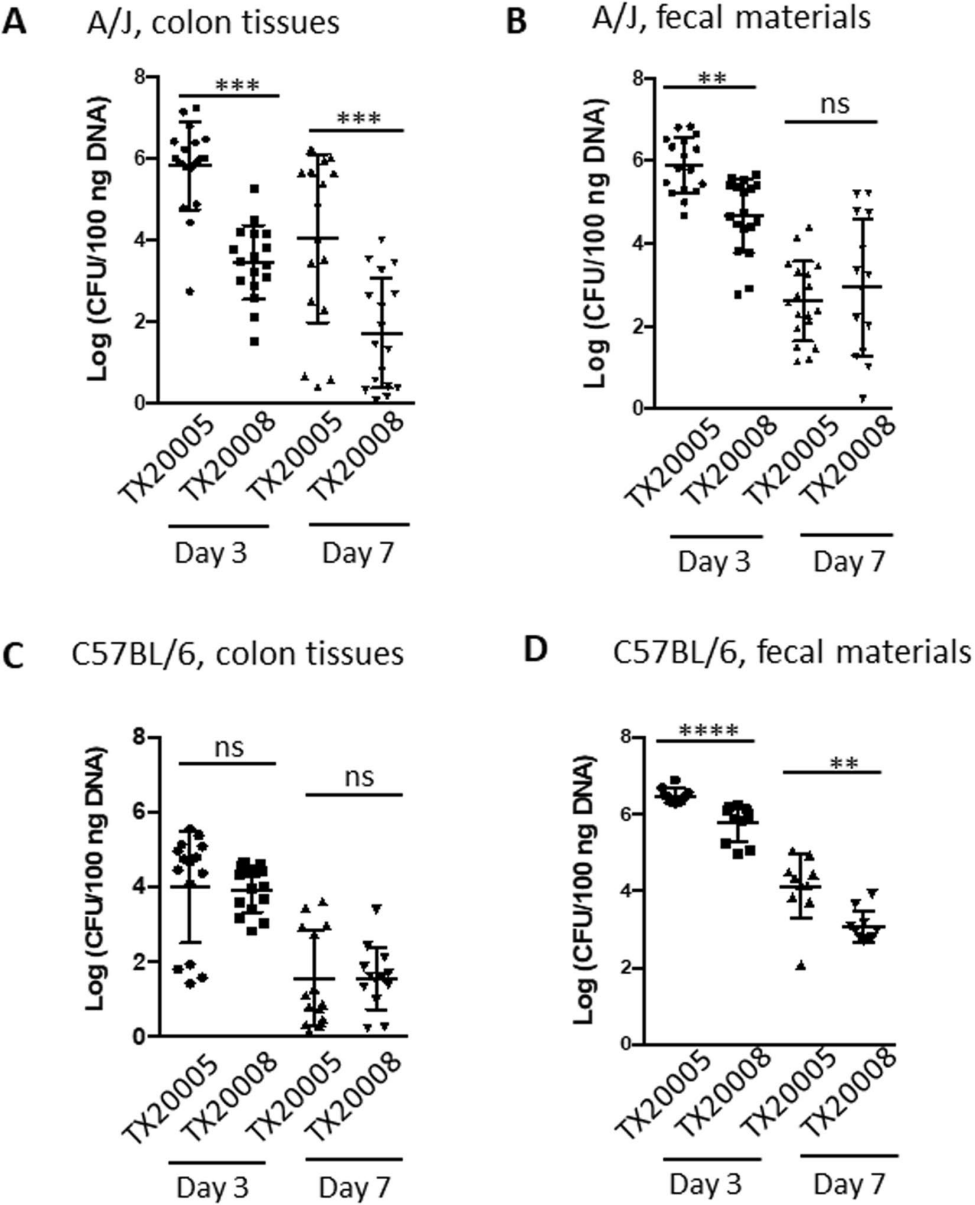
Colonization of the mouse colon by PP-*Sg* and NP-*Sg* strains. A/J (A and B) and C57BL/6 (**C** and **D**) mice were orally gavaged with *Sg* bacteria as described in the Methods section. Colon tissues (**A** and **C**) and fecal materials (**B** and **D**) were harvested at day 3 and 7 post oral gavage. *Sg* titer was determined using qPCR as described in the Methods section. **p < 0.01; ***p < 0.001; ****p < 0.0001; non-parametric Mann-Whitney test.

Mice gavaged with TX20005 had significantly higher mean *Sg* concentration in colon tissues than mice gavaged with TX20008 at day 3 (~182-fold, *p* < 0.0001) and day 7 (~307-fold, *p* = 0.0009), respectively (Fig. 5A). In fecal materials, mice gavaged with TX20005 also had significantly higher mean *Sg* concentration than those with TX20008 at day 3 (~13-fold, *p* = 0.0002), although the difference was much smaller than that in colon tissues. At day 7, however, there was no statistically significant difference in the *Sg* concentration between the two groups (*p* = 0.6079) (Fig. 5B). These results suggest that TX20005 is better at colonizing the mouse colon than TX20008, and that the ability to colonize colon tissues is the primary difference between TX20005 and TX20008 in colonizing the colon of A/J mice.

The *Sg* genome contains genes encoding multiple pili^30–32^. The Pil1 locus encodes a collagen adhesion Acb that can potentially mediate bacterial adherence to host tissues^32^. The Pil3 locus was reported to be important for *Sg* to adhere to colonic mucus and to colonize the mouse gut^33^. We examined if the difference between TX20005 and TX20008 in colonizing colon tissues could be due to the absence or lack of expression of Pil1 or Pil3 in the latter strain by using PCR and RT-PCR. The results showed that both strains express Pil1A and Pil3A at comparable levels by RT-PCR (Supplemental Fig. 3), suggesting that other TX20005-specific factors likely contribute to the increased ability of this organism to colonize colon tissues.

### Host factors contribute to gut colonization by *Sg*

We also examined gut colonization in C57BL/6 mice. In contrast to the results from A/J mice, TX20005 appears to not have any advantage over TX20008 at colonizing colon tissues in C57BL/6 mice. There was no significant difference in the *Sg* concentration in colon tissues between the TX20005 and the TX20008 groups at either day 3 (*p* = 0.1251) or day 7 (*p* = 0.6083) (Fig. 5C). To further examine this, we directly compared the *Sg* concentration in the colon tissues between A/J and C57BL/6 mice. TX20005 associated with colon tissues of A/J mice significantly better than with C57BL/6 mice at both day 3 (~40-fold, *p* < 0.0001) and 7 (~530-fold, *p* = 0.0019) (Fig. 5A and C). On the other hand, we observed no significant difference in the *Sg* concentration in the colon tissues between A/J and C57BL/6 mice gavaged with TX20008 at either day 3 (*p* = 0.0964) or 7 (*p* = 0.8875). This suggests that TX20005 colonizes colon tissues better than TX20008 only in A/J mice, suggesting that specific host factors/structures on the colonic epithelium of A/J mice contribute to TX20005 colonization of colon tissues. In fecal materials, the TX20005 group had significantly more *Sg* bacteria than the TX20008 group at both day 3 (*p* < 0.0001) and 7 (*p* = 0.0029). This suggests that TX20005 is still better than TX20008 at surviving in the colon of C57BL/6 mice. The difference in the ability of TX20005 to colonize colon tissues between A/J and C57BL/6 appears not to be due to the ability of the organism to survive in the colon of the latter group of mice. This is because we observed higher TX20005 concentration in fecal materials from C57BL/6 than those from A/J (Fig. 5B and D). Taken together, these results suggest that not only are there variations among *Sg* strains in the ability to colonize mouse colon, but the host genetic background also contributes the ability of *Sg* strains to attach to colon tissues.

### NP-*Sg* strain TX20008 does not promote tumor development in a mouse model of CRC

**Figure 6:**
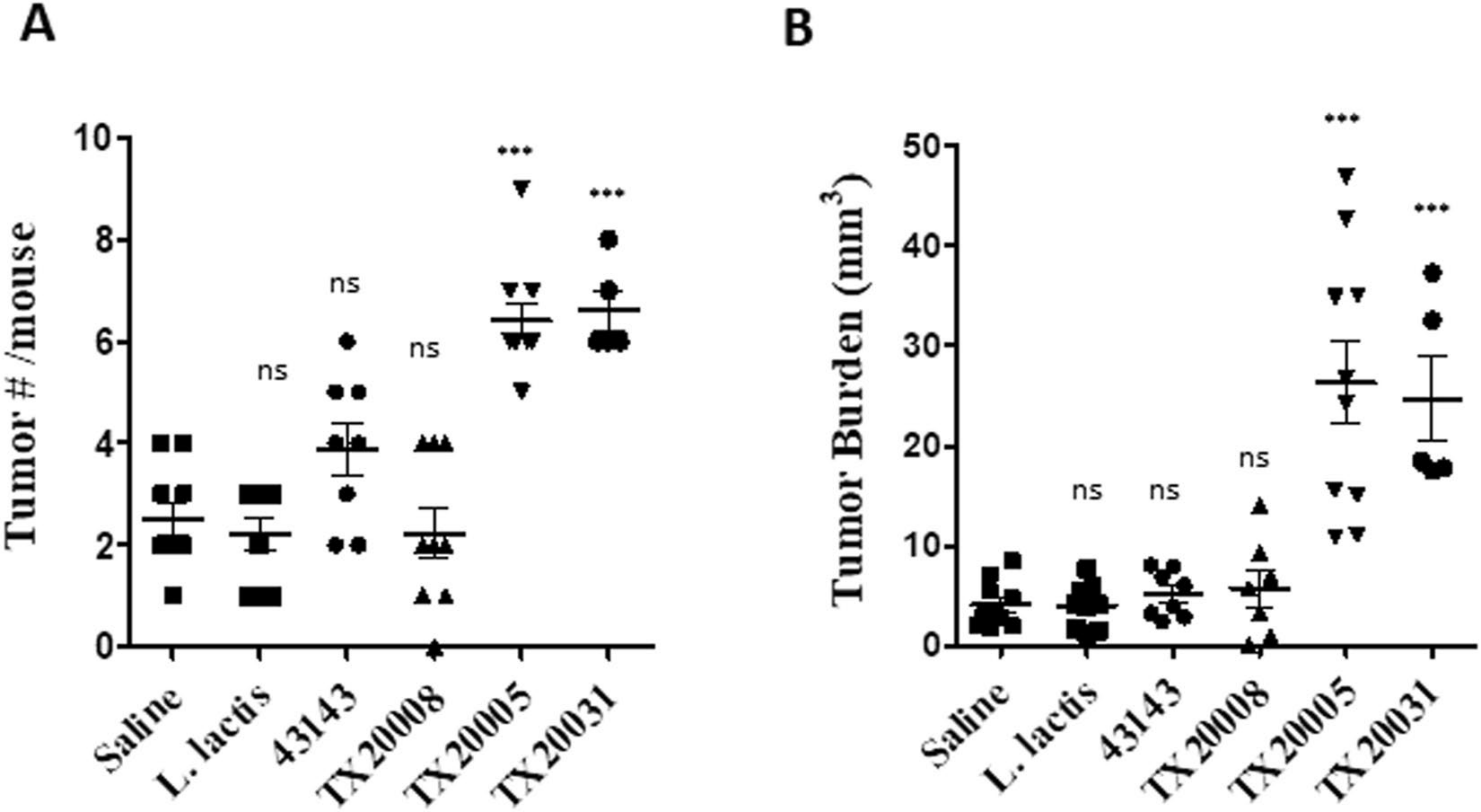
The effect of PP-*Sg* and NP-*Sg* strains on colon tumor development. A/J mice were injected with AOM, treated with antibiotics, and orally gavaged with saline, *L. lactis*, TX20005, TX20031, TX20008 or ATCC 43143, as described in the Methods section. Colon was examined visual for the presence and size of tumors by blinded observers. ***p < 0.001; non-parametric Mann-Whitney test, vs. saline control.

It was previously reported that TX20005 is able to promote colon tumor development in an AOM-induced mouse model of CRC^10^. We further examined the effect of TX20031, TX20008 and ATCC 43143 on colon tumor development in this model. AOM-treated mice were given 12 weekly gavages of TX20005, TX20031, TX20008, ATCC 43143, *L. lactis*, or saline. Colon was evaluated macroscopically for tumor number and size in a blind fashion. We observed that mice treated with TX20005 had significantly more tumors (Fig. 6A) and higher tumor burden (Fig. 6B) compared to the saline or *L. lactis* group (*p* < 0.0001), as expected. TX20031 showed a similar effect as TX20005. Mice treated with TX20031 exhibited significantly higher tumor number and tumor burden compared to the saline or *L. lactis* control group. In contrast, exposure to TX20008 or ATCC 43143 had no effect on tumor number or burden compared to the saline or *L. lactis* group. These results suggest that the NP-*Sg* strains were not able to promote colon tumor development in this model system.

## Discussion

A strong correlation between *Sg* and CRC has been reported for several decades. *Sg* was recently demonstrated to actively promote colon tumor development^10^, suggesting that *Sg* is also functionally important to CRC. More studies are necessary to gain a deeper understanding of the specific details of the *Sg*-CRC association. In this report, we showed that there are variations among *Sg* strains with respect to their ability to promote target host cell proliferation, to adhere to host cells, to colonize the colon and to promote colon tumor development. In addition, our results suggest that the ability of *Sg* to colonize colon tissues is affected by specific host factors. These data have important implications with respect to clinical correlation studies in the future as well as the mechanism underlying the tumor-promoting activity of *Sg*.

We found that some *Sg* strains are able to promote host cell proliferation while others are not. This is determined by examining viable cell numbers and the level of cell proliferation markers from co-culture experiments *in vitro*, and by comparing tumor size between cells treated with PP-*Sg* and NP-*Sg* respectively, in a xenograft model *in vivo*. These results suggest that strain-specific bacterial factors are involved in its stimulation of host cell proliferation. It was found previously that close contact between *Sg* TX20005 and colon cancer cells was necessary for the organism to stimulate cell proliferation^10^. Here we observed that the adherence of NP-*Sg* strains to target host cells was significantly lower when compared to PP-*Sg* strains. This is consistent with previous results and provides further support that direct contact between *Sg* bacteria and host cells is important for it to stimulate cell proliferation. The observation that neither stationary or exponential phase cultures of NP-*Sg* strains were able to stimulate host cell proliferation suggests that the defective phenotype of these strains was not due to different regulation of gene expression by growth phase. It is possible that surface factors on PP-*Sg*, which are absent or inactive in NP-*Sg*, mediate the initial interaction with a host cell receptor. This then triggers downstream signaling events leading to host cell proliferation. Alternatively, adherence to host cells “anchors” the bacteria on the cell surface, thereby bringing other secreted or soluble factors in close proximity to the host cell surface at relatively high concentrations and allowing them to exert their effect on host cell proliferation. Genomic and transcriptome comparison between PP-*Sg* and NP-*Sg* strains are currently on-going to identify PP-*Sg*-specific factors.

NP-*Sg* strain TX20008 was significantly less effective at colonizing the colon tissues of A/J mice compared to PP-*Sg* strain TX20005 at both day 3 and 7 post oral gavage of the bacteria. In fecal materials on the other hand, although there is a difference in *Sg* concentration at day 3, the difference disappeared by day 7. This suggests that at least at the longer time point, the lower TX20008 concentration in colon tissues is unlikely due to the ability of the organism to survive in the colon environment. This, combined with the results from cell adherence assays, suggests that adherence to colonic epithelial cells contributes to *Sg* colonization of colon tissues *in vivo*.

In the AOM-CRC model in A/J mice, TX20008 and ATCC 43143 showed no effect on tumor number or tumor burden as compared to mice treated with saline or the negative control bacteria, in contrast to the results for TX20005 and TX20031. This suggests that attachment to colon tissues through interactions between specific *Sg* factors and colonic epithelial receptors/structures may be the first necessary step for the tumor-promoting activity of *Sg*.

The observation that the advantage of TX20005 in colonizing colon tissues over TX20008 is only observed in A/J but not in C57BL/6 mice is interesting. This suggests that the ability of *Sg* to colonize colon tissues is influenced by the host genetic background, further pointing towards interactions between specific bacterial and host factors. Identifying these specific bacterial and host factors will be key to understanding the mechanism underlying the *Sg*-CRC association. The results described here also have implications in the selection of animal models in studies to investigate the biological activities of *Sg* in the colon.

*Sg* previously belonged to the *S. bovis* group of organisms. Based on biochemical characteristics such as the ability to ferment mannitol, *S. bovis* was then split into three biotypes: biotype I, biotype II/1 and biotype II/2^34,35^. The group was later reclassified into several species and subspecies based on molecular and DNA analyses. Biotype I now corresponds to *S. gallolyticus* subsp. *gallolyticus* (*Sg*), biotype II/1 consists of *S. infantarius* subsp. *infantarius* and *S. lutetiensis* (also called *S. infantarius* subsp. *coli*), and biotype II/2 consists of *S. gallolyticus* subsp. *pasteurianus* and *S. gallolyticus* subsp. *macedonicus*^36^. Earlier surveys or case studies did not distinguish the different species and subspecies within the *S. bovis* group. It was later recognized that only *Sg* within the *S. bovis* group displayed a strong association with CRC. For example, patients with *Sg* infections have ~7 fold higher risk of having concomitant colorectal adenoma/adenocarcinoma compared to patients infected with other species within the *S. bovis* group^13^. In CRC patients, the prevalence of *Sg* in tumor tissues (~74%) is also much higher than that of *S. gallolyticus* subsp. *pasteurianus* (~7%)^10^. It became clear that distinguishing the various species and subspecies within the *S. bovis* group is necessary for gaining a proper understanding of the correlation between these bacteria and the clinical, and pathological characteristics of CRC. In this context, our results further suggest that clinical correlation studies may need to go even beyond the subspecies level to the specific strains. This will require more extensive studies to identify specific *Sg* factors that can serve as molecular markers.

In conclusion, this is the first report on variations among *Sg* clinical isolates with respect to host cell adherence, stimulation of cell proliferation, colonization of colon tissues of mice and promotion of colon tumor development. These findings provide hints at how *Sg* functions to promote tumor development and have important implications to clinical correlation studies. Investigations to identify specific *Sg* and host factors involved in these activities will be important.

## Methods

### Bacterial strains, cell lines and culture conditions

*Sg* strains TX20005, TX20008, TX20030, TX20031, and TX20034 were kindly provided by B. E. Murray, University of Texas Health Science Center, Houston, TX. They were isolated from the blood of endocarditis patients^29^. ATCC 43143 was purchased from ATCC. *Sg* strains and *Lactococcus lactis* MG1363 were grown at 37 °C in brain-heart infusion (BHI) broth with shaking or on BHI or Tryptic Soy agar plates (Difco Laboratories, Sparks, MD), as previously described^10^. Stationary phase bacteria were harvested after 16 hours of culturing. Exponential phase bacteria were obtained by diluting overnight culture into fresh media (1:100) and cultured for 6 hours.

Human colon cancer cells lines HCT116 and HT29 were cultured in Dulbecco’s Modified Eagle’s Medium supplemented with 10% fetal bovine serum (FBS) (GIBCO, USA).

### Cell proliferation assay

This was performed as previously described^10^. Briefly, stationary or exponential phase bacteria were resuspended in cell culture media and added to cells in tissue culture plates (multiplicity of infection = 0.01) for 24 hours. Cells were then detached by trypsin treatment, stained with trypan blue and enumerated in a Cellometer® Mini automated cell counter (Nexcelome Biosciences, Lawrence, MA).

### Western blot assays

This was performed as described previously^10^. Briefly, cells were co-cultured with bacteria or media only for 12 hours and washed with PBS for three times. Nuclear proteins were extracted. Nuclear lysates were subjected to SDS-PAGE and western blot. Rabbit polyclonal antibodies against β-catenin (1:4000), c-Myc (1:3000), PCNA (1:2000), β-actin (1:5000) and lamin B1 (1:1000) were all from Cell Signaling Technology (CST). Horse radish peroxidase (HRP)-conjugated anti-rabbit IgG (CST) was used as the secondary antibody. Signals were detected using HyGLO, chemiluminescent HRP (Denville, Mteuchen, NJ). Band intensity was quantified using Image J.

### Adherence assay

This was performed following a procedure described previously^10^. Briefly, cells were seeded onto the wells of 24-well tissue culture plates. Stationary phase bacteria were resuspended in DMEM supplemented with 10% FBS, and added to the wells at a multiplicity of infection (MOI) of 10. The plates were incubated in a humidified incubation chamber at 37 °C with 5% CO_2_ for 1 hour. After washing, cells were lysed with sterile PBS containing 0.025% Triton X-100 and dilution plated onto BHI agar plates to determine the number of adhered bacteria.

### Animal experiments

Animal studies were performed in accordance with protocols approved by the Institutional Animal Care and Use Committee at the Texas A&M Health Science Center, Institute of Biosciences and Technology.

The xenograft experiment was performed as described previously^10^. Briefly, HCT116 cells were incubated with TX20005 or TX20008 (MOI = 1) for 12 hours. The cells were washed, trypsinized, mixed with Matrigel (Corning, MA) and subcutaneously injected into nude mice (Jackson Laboratory, Bar Harbor, ME). Three hours after the injection, mice were administered a broad-spectrum antibiotic imipenem (MSD) by intraperitoneal (i.p.) injection (150 mg/kg body weight). Tumor diameters were measured with a digital caliper, and tumor volume calculated.

For gut colonization, eight-week old female A/J or C57bl/6 mice (Jackson Laboratory, Bar Harbor, ME) were treated with ampicillin (1 g/L) in drinking water for one week and then switched to water without the antibiotic. 24 hours later, mice were orally gavaged with TX20005 or TX20008 (~1 × 10^9^ cfu/mouse). Mice were euthanized after 3 or 7 days post gavage. Colon and fecal materials were collected and frozen at −80 °C until DNA extraction.

The AOM-induced mouse model of CRC was performed as described previously^10^ with slight modifications. Briefly, eight-week old A/J mice (Jackson Laboratory, Bar Harbor, ME) were treated with 4 weekly injections of AOM (10 mg/kg body weight, i.p.), followed by one week of ampicillin (1 g/L) in drinking water. Mice were orally gavaged with saline, or bacteria once a week for 12 weeks and euthanized one week after the final gavage. Colons were visually examined for macroscopic tumors and tumor size measured using a digital caliper. Visual evaluation was carried out by blinded observers.

### Detection of *Sg* in colon tissues and fecal materials by qPCR

QIAamp Fast DNA Stool Mini Kit and AllPrep DNA/RNA/Protein Mini Kit (Qiagen) were used to extract DNA from fecal materials and colon tissues, respectively. qPCR was performed following a protocol described previously^10^. Briefly, qPCR was performed using Fast Plus EvaGreen qPCR Master Mix (Biotium) in a Viia 7 Real Time PCR System (Applied Biosystems). ΔCT was normalized to the results using universal 16S rRNA primers. DNA was also extracted from mouse colon tissues from healthy uninfected mice spiked with known concentrations of serially diluted *Sg* bacteria. These DNA templates were included in each round of qPCR experiment to generate a standard curve. The concentration of *Sg* in fecal materials and colon tissues was then determined by performing linear regression analysis (GraphPad Prism 6).

### Genomic DNA, RNA extraction from *Sg* and RT-PCR

Genomic DNA was extracted from *Sg* strains by using QIAamp DNA Mini Kit (Qiagen) following the instructions by the supplier. To extract RNA from *Sg* strains, bacteria were collected from stationary phase and RNA isolated by using RNeasy kit (Qiagen). Reverse transcription was performed using ProtoScript® First Strand cDNA Synthesis Kit (NEB). Pil1A and Pil3A was detected using the following primer sequences: Pil1AF, TGAGAACGGTGGTAGTGGAAC; Pil1AR, 5′TCTTCTGCT TGGCTTGCAGT; Pil3AF, CCGTCTGCTTCTGTGAAGGT; Pil3AR: AAGCGTCAACAT CCGTTCCT.

## Electronic supplementary material

Supplemental Information
