# Supplementary material for: Variations among Streptococcus gallolyticus subsp. gallolyticus strains in connection with colorectal cancer
Source: Sci Rep. 2018 Jan 24;8:1514. doi: 10.1038/s41598-018-19941-7 (PMC5784120; doi:10.1038/s41598-018-19941-7)
Supplement: Supplementary file 1 — Supplemental Information [file 41598_2018_19941_MOESM1_ESM.pdf]

Variations among *Streptococcus gallolyticus* subsp. *gallolyticus* strains in connection with colorectal cancer

Ritesh Kumar[1#], Jennifer L. Herold[1#], John Taylor[1], Juan Xu[1], and Yi Xu[1*]

[1] Center for Infectious and Inflammatory Diseases, Institute of Biosciences and Technology, Texas A&M Health Science Center, Houston, TX

#These authors contributed equally to this work.

*Corresponding author:

Yi Xu

Center for Infectious and Inflammatory Diseases, Institute of Biosciences and Technology, Texas A&M Health Science Center

2121 W. Holcombe Blvd.

Houston, TX 77030

E-mail: yxu@ibt.tamhsc.edu

Supplemental Figure 1. PP-*Sg* and NP-*Sg* differ in their ability to up-regulate cell proliferation markers. HT29 cells were co-cultured with PP-*Sg* strains TX20030 and TX20031, NP-*Sg* strains TX20034 and ATCC 43143, *L. lactis* or media only as described in the Methods section. Nuclear extracts were subjected to SDS-PAGE and western blot. Representative images are shown (**A**). Band intensities were measured using Image J and normalized to that of lamin B1 first, and then to cells only control. Data shown were combined from at least three independent experiments (**B**). *, $p < 0.05$; **, $p < 0.01$; unpaired two-tailed t test, vs. cells only.

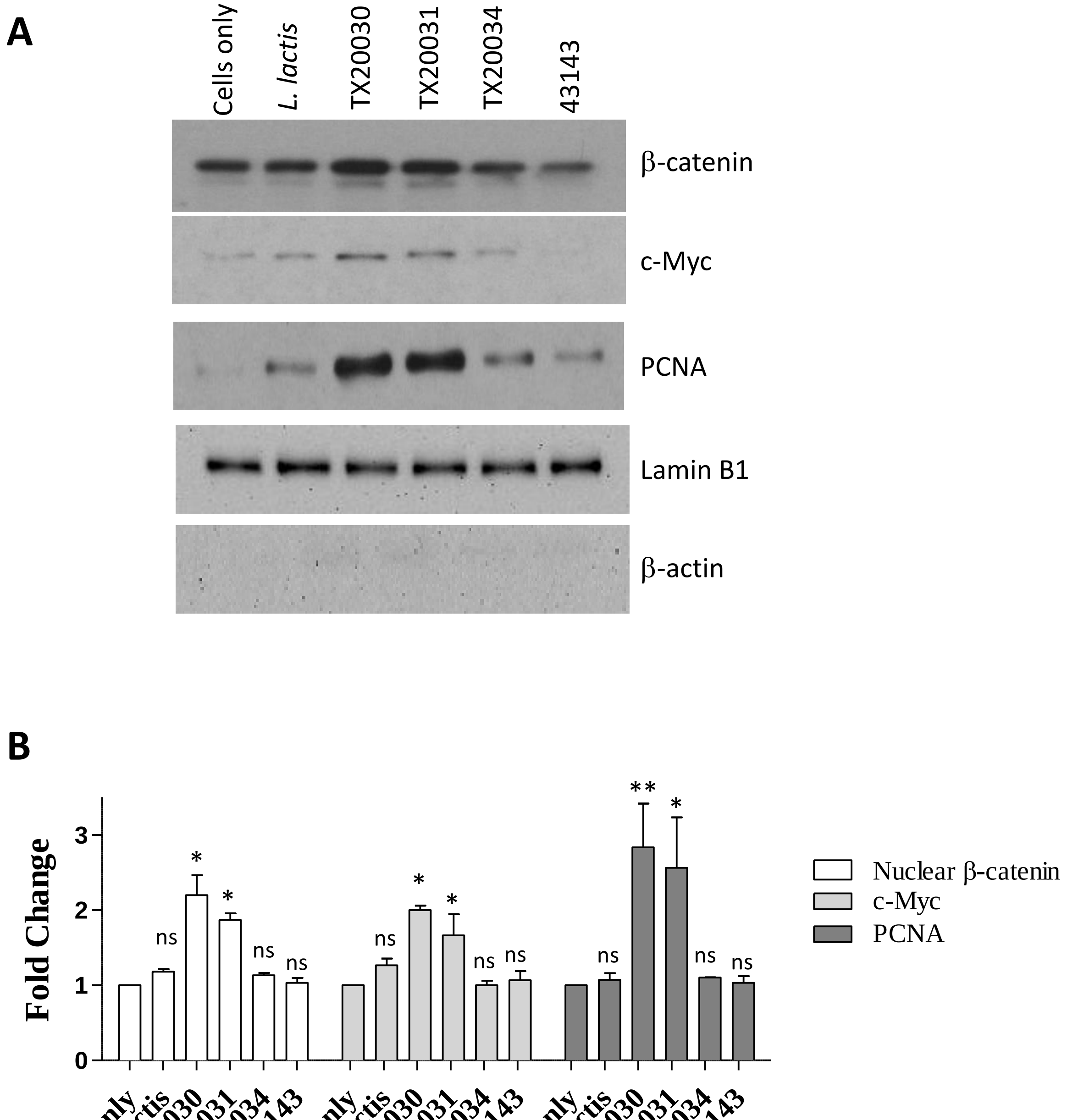

Supplemental Fig. 1

Supplemental Figure 2. Effect of stationary and exponential phase bacteria on cell proliferation. Bacteria harvested at exponential or stationary phase of growth were added to HCT116 cells and co-cultured for 24 hours. Viable cell numbers were enumerated. The cell numbers were normalized to no bacteria control. The results shown were combined from at least three independent experiments, each done with two technical replicates. *, $p < 0.05$; **, $p < 0.01$; ***, $p < 0.001$, unpaired two-tailed t test.

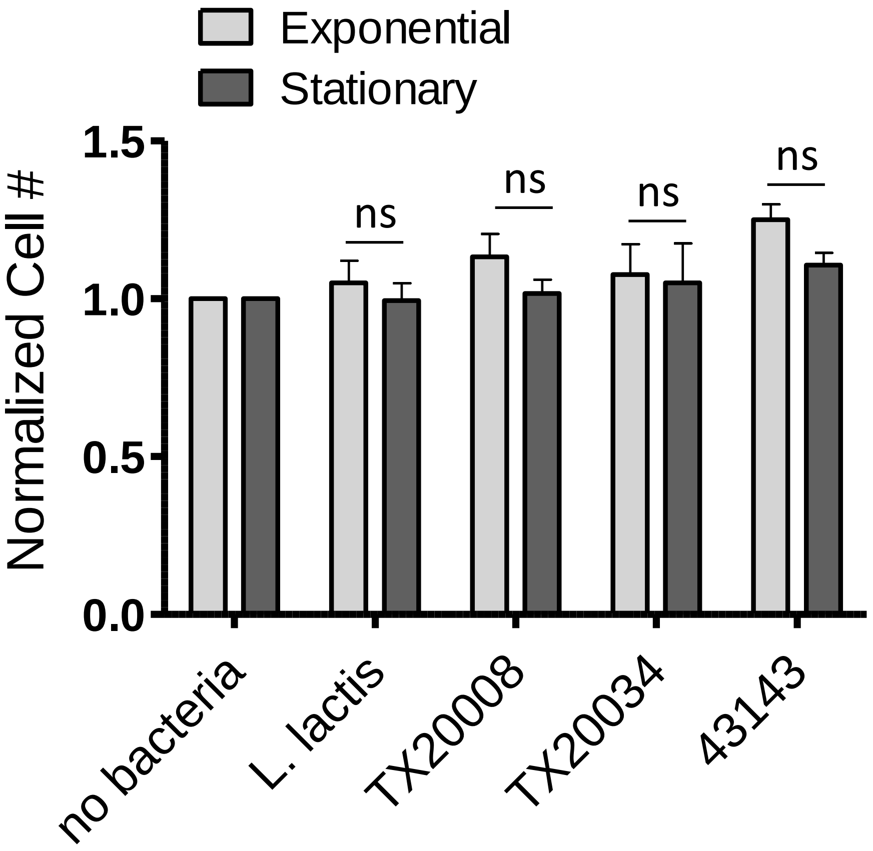

Supplemental Fig. 2

Supplemental Figure 3. TX20005 and TX20008 express Pil1A and Pil3A at similar levels. Genomic DNA was isolated by using QIAamp DNA Mini Kit (Qiagen). RNA was isolated from stationary cultures and cDNA synthesized as described in the Methods section. Specific primers for PilA and Pil3A genes were used for PCR amplification using genomic DNA, cDNA and RNA as templates.

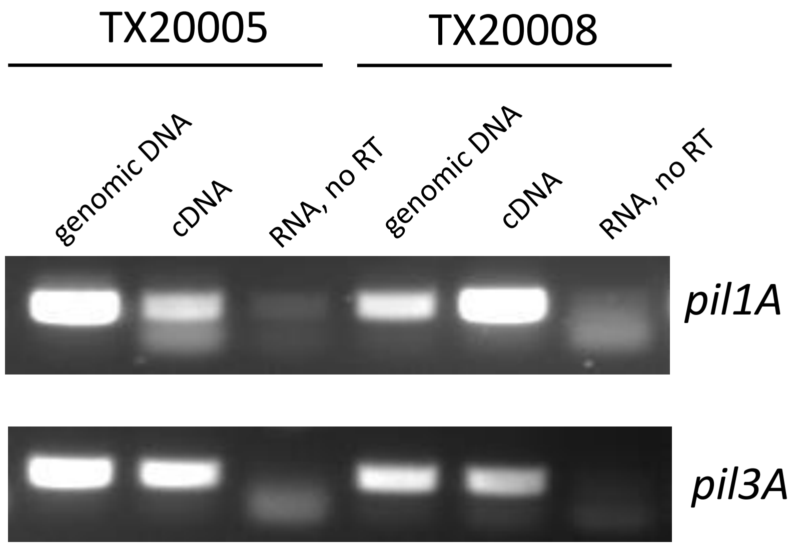

Supplemental Fig. 3

Full length blots
Figure 2

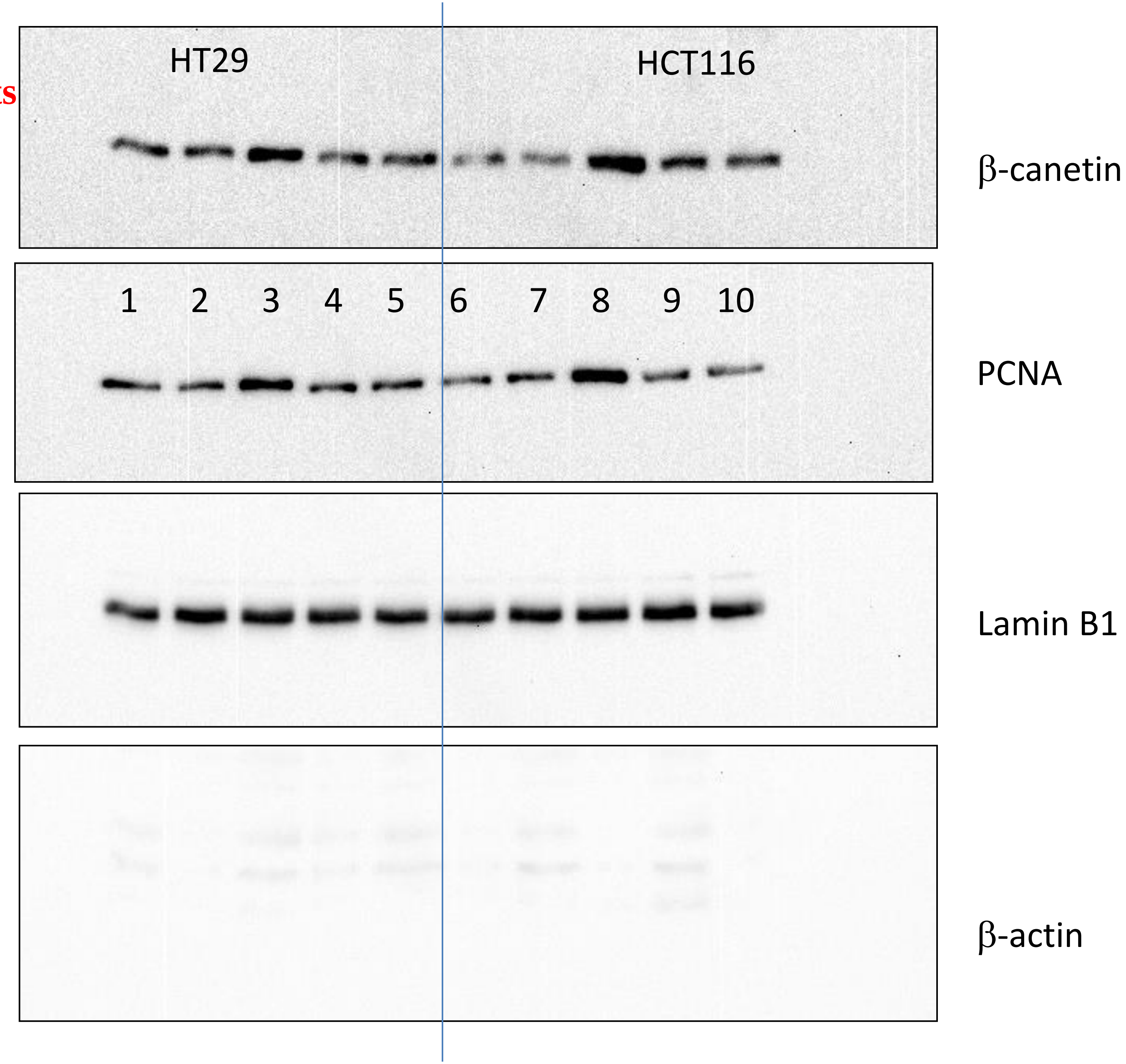

Lanes 1 to 4 are used in Figure 2A and lanes 6 to 9 are used in Figure 2C

Full length blots
Figure 2

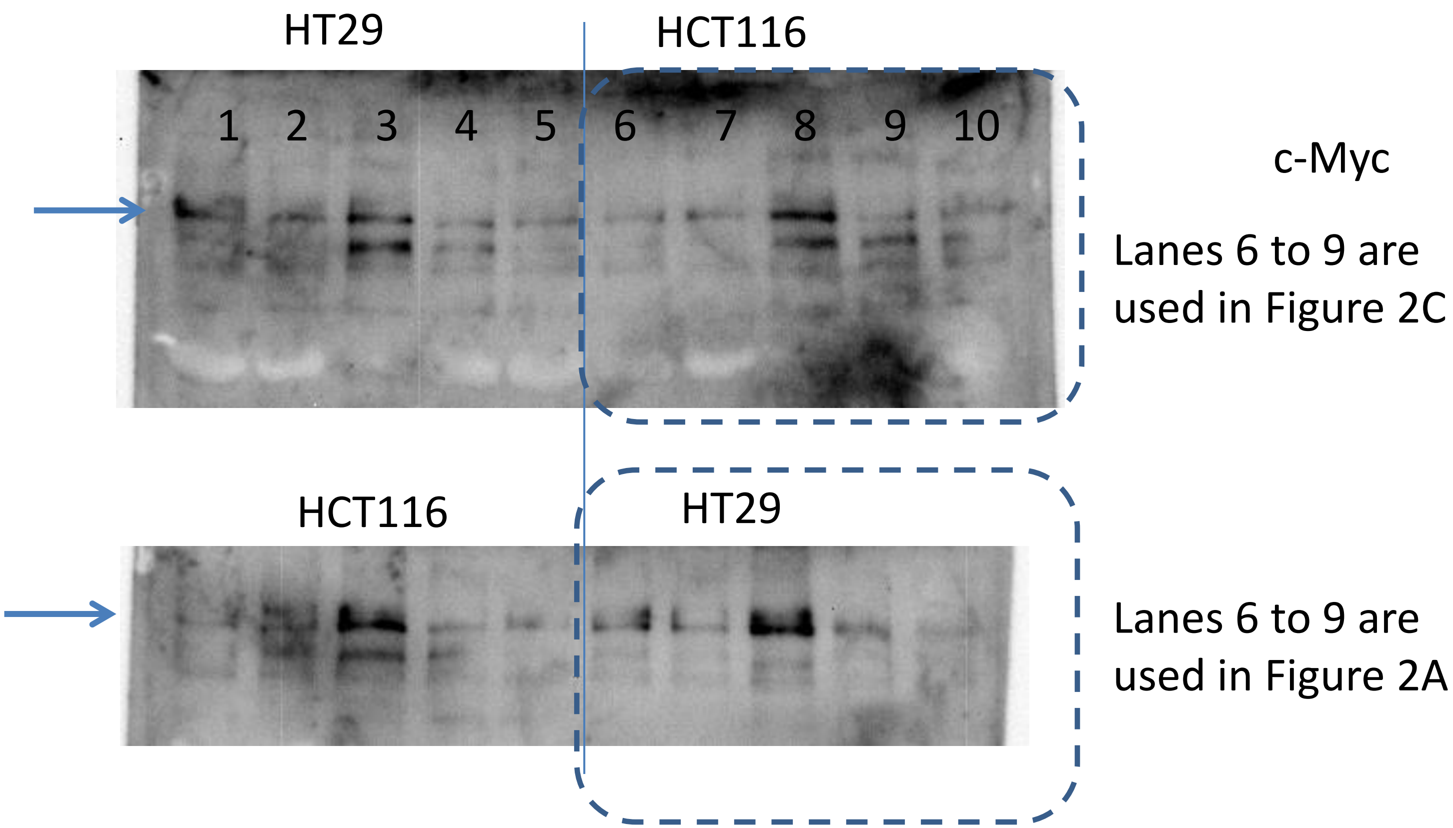

Full length blots for Supplemental Fig 1

Lane No	
1	No bacteria
2	*L.lactis*
3	TX20030
4	TX20031
5	TX20034
6	43143

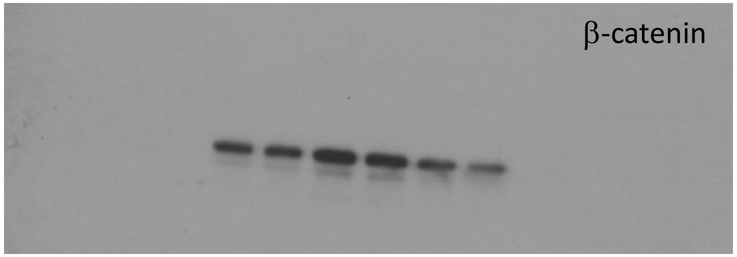

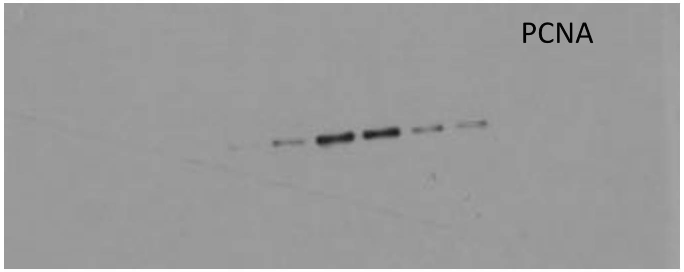

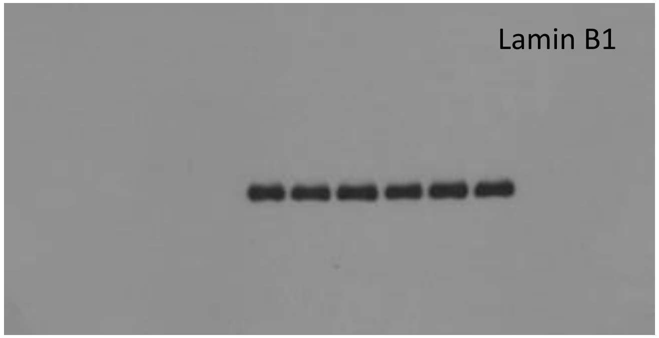

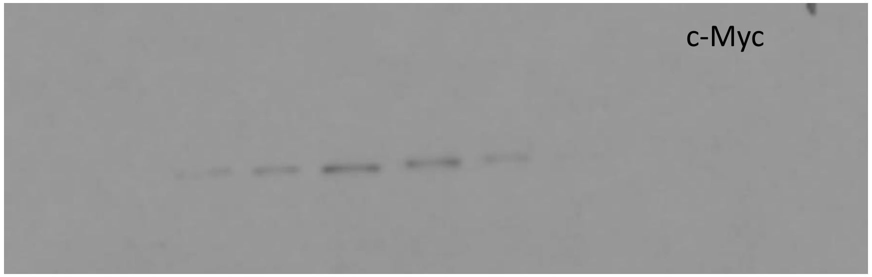

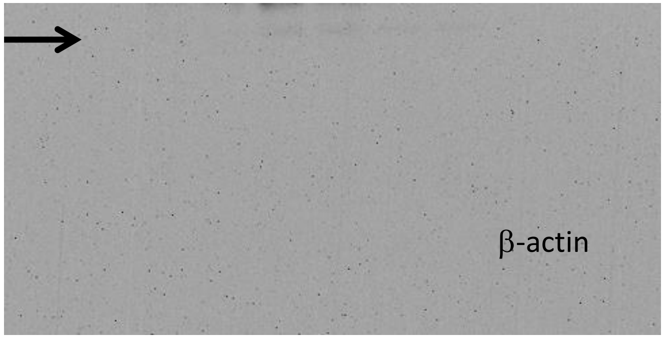

Full length gels
Supplemental Figure 3

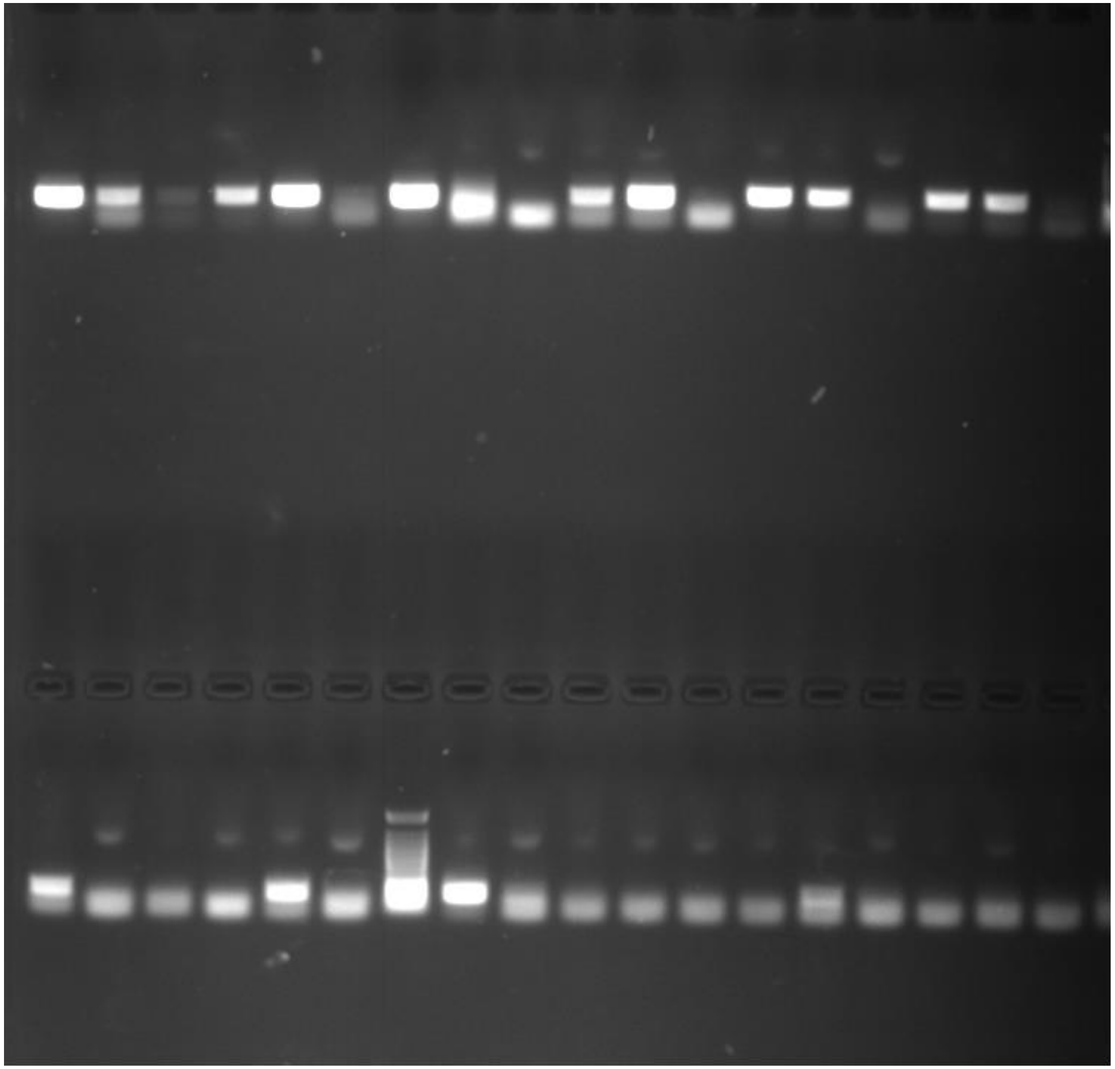

Top panel, lanes 1-6 and 13-18 are used in Supplementary Figure S1.
Lanes 1-3 are TX20005 pilA, 4-6 are TX20008 pil1A
Lanes 13 – 15 are TX20005 pil3A, 16-18 are TX20008 pil3A
